# Cell-Free Urinary MicroRNA-99a and MicroRNA-125b Are Diagnostic Markers for the Non-Invasive Screening of Bladder Cancer

**DOI:** 10.1371/journal.pone.0100793

**Published:** 2014-07-11

**Authors:** Ding-Zuan Zhang, Kin-Mang Lau, Eddie S. Y. Chan, Gang Wang, Cheuk-Chun Szeto, Kenneth Wong, Richard K. W. Choy, Chi-Fai Ng

**Affiliations:** 1 Division of Urology, Department of Surgery, The Chinese University of Hong Kong, Hong Kong SAR, China; 2 Department of Anatomical and Cellular Pathology, The Chinese University of Hong Kong, Hong Kong SAR, China; 3 Division of Nephrology, Department of Medicine and Therapeutic, The Chinese University of Hong Kong, Hong Kong SAR, China; 4 Department of Obstetrics & Gynaecology, The Chinese University of Hong Kong, Hong Kong SAR, China; UCSF / VA Medical Center, United States of America

## Abstract

**Background:**

Evidence implicated the diagnostic significance of microRNAs in whole urine/urine sediments in urothelial carcinoma of the bladder (UCB). However, the contaminated blood cells in patients with haematouria significantly altered the expression profiles of urinary microRNA, influencing the test accuracy.

**Methods:**

MicroRNA profiles of the urine supernatants of UCB patients and controls without any malignancy and profiles of malignant and corresponding normal mucosa tissues from the patients were determined by microRNA microarray and compared to identify differentially expressed microRNAs. The differential expression was verified in the tissues of an independent patient cohort by RT-qPCR. The diagnostic significance of selected microRNAs as biomarkers in the urine supernatant was investigated in the expanded cohorts.

**Results:**

MicroRNA-99a and microRNA-125b were down-regulated in the urine supernatants of UCB patients. The degree of down-regulation was associated with the tumor grade. A diagnostic model was developed using a combined index of the levels of microRNA-99a and microRNA-125b in the urine supernatant with a sensitivity of 86.7%, a specificity of 81.1% and a positive predicted value (PPV) of 91.8%. Discriminating between high- and low-grade UCB, the model using the level of microRNA-125b alone exhibited a sensitivity of 81.4%, a specificity of 87.0% and a PPV of 93.4%.

**Conclusions:**

The results revealed a unique microRNA expression signature in the urine supernatants of UCB patients for the development of molecular diagnostic tests. An effective cell-free urinary microRNA-based model was developed using a combined index of the levels of microRNA-99a and microRNA-125b to detect UCB with good discriminating power, high sensitivity and high specificity.

## Introduction

Urothelial carcinoma of the bladder (UCB) is the second most common malignancy in the urinary system [1]. Due to its high incidence and frequent recurrence, effective diagnostic and disease monitoring tools are essential for the clinical management of patients. Cystoscopy is currently the standard clinical test for diagnosis and cancer surveillance. However, the procedure is invasive and unpleasant, and has several practical limitations. For example, it may not be able to detect small and/or flat tumors like carcinoma *in situ*. Therefore, an alternative non-invasive approach exhibiting high specificity and sensitivity is required. Although many blood- and urine-based biomarkers have been identified and evaluated in the literature, none have been ideal and powerful enough to replace cystoscopy [2].

MicroRNAs, which are small non-coding RNAs, have recently demonstrated significant diagnostic and prognostic value in various types of cancer [3]–[5]. MicroRNAs exhibit high stability and easy detectability even at low levels in various types of clinical samples [6]–[8]. It is considered an excellent disease biomarker for detection and monitoring. One study demonstrated that a number of microRNAs were aberrantly expressed in UCB tumor tissues and that these aberrations could be engaged in diagnosis and the staging of bladder cancer biopsies [9]. In addition, the assessment of the levels of these aberrantly expressed microRNAs that show unique signatures in whole urine or urine exfoliated cells is even more promising for the diagnosis and disease surveillance of UCB [10]–[11]. However, the results are sometimes unreliable when studying patients with haematuria, in whom contaminated blood cells significantly alter the urinary microRNA profiles and thus mask the signatures. In fact, 85% of patients exhibit varying degrees of haematuria [12]–[13]. As a result, an alternative approach to measuring microRNAs is required. Evidence has suggested that free nucleic acids, such as DNA biomarkers in urine supernatants, provide a higher detection rate and higher sensitivity than those in sediment for UCB diagnosis [14]–[16]. Therefore, assessment of the levels of microRNAs in urine supernatant could improve the use of microRNAs as diagnostic biomarkers for detecting UCB, especially in patients with haematuria. In this study, the feasibility of using cell-free urinary microRNAs for diagnosing UCB was determined and models for diagnosing UCB and discriminating tumor grades were developed.

## Methods

### Patients and samples

An overview of this study is shown in Figure S1. With the written consent of the donors and the approval of the Joint Chinese University of Hong Kong - New Territories East Cluster Clinical Research Ethics Committee, tissue and urine samples were collected in the Urology Unit, Department of Surgery, Prince of Wales Hospital, Hong Kong. Mid-stream urine was collected from bladder cancer patients before surgery. Both tumor tissue and normal bladder mucosa located >3 cm away from the tumor edge were obtained by cystoscopy. The 1973 WHO diagnosis and grading system for bladder cancer [17] was used for diagnosis. For the normal controls, urine samples were collected from patients who had normal cystoscopic findings, absence of malignancies with a >6 months follow-up and haematuria. All of the urine samples were centrifuged at 2,500 r.c.f. for 20 minutes and the urine supernatants were collected.

### MicroRNA microarray

The total RNA of the urine supernatants and frozen tissue was extracted using the MirVanaPARIS Kit (Ambion) in accordance with the manufacturer's recommended protocols. The Agilent Human miRNA Microarray Chip (Release 13.0, Agilent Technologies, Santa Clara, CA, U.S.A.), which encompasses 866 human microRNAs and 89 viral microRNAs, was used to profile the expression of these microRNAs in the urine supernatants and cancer and control tissues. The dataset was deposited to ArrayExpress (https://www.ebi.ac.uk/arrayexpress/) and the accession number is E-MTAB-2573. The signal intensity of each spot was median-normalized and further normalized by linear regression.

### Reverse transcription-quantitative polymerase chain reaction (RT-qPCR)

First strand cDNA was synthesized from the total RNA of the urine supernatants and tissue samples using a universal cDNA synthesis kit (Exiqon, Vedbaek, Denmark) in accordance with the manufacturer's recommended protocol. The resultant cDNA was subjected to quantitative polymerase chain reaction (qPCR) with SYBR Green master mix and microRNA LNA PCR primer (Exiqon) that specifically recognized the targeted microRNA in an ABI 7900HT fast RT-qPCR machine (Applied Biosystem/Life Technologies, Grand Island, NY, U.S.A.). RNU6B was used as the reference control. All of the samples were tested in duplicate. The relative level of microRNA was determined using the ΔΔC_q_ method.

### Statistical methods

The microarray data were analyzed using the Gene Spring 11.0 software (Agilent) to normalize and identify the differentially expressed microRNAs. ABI SDS 2.3 software (ABI/Life Technologies) was used to analyze the RT-qPCR data. The quantitative data were subjected to a Mann-Whitney U test and a Wilcoxon signed-rank test using the SPSS 16.0 software (IBM Co, Armonk, NY, U.S.A.). In subsequent post-hoc analysis, a bivariate logistic regression analysis was performed to determine the Combined Index (CI) for the combination of two microRNAs expression levels. A receiver operating characteristic (ROC) curve analysis was carried out using the SPSS 16.0 software. The sensitivity, specificity, positive and negative predictive values, and positive and negative likelihood ratios were calculated.

## Results

### Identification of differentially expressed microRNAs between the urine supernatants of UCB patients and controls and between cancer and normal tissues by microRNA microarray

**Figure 1:**
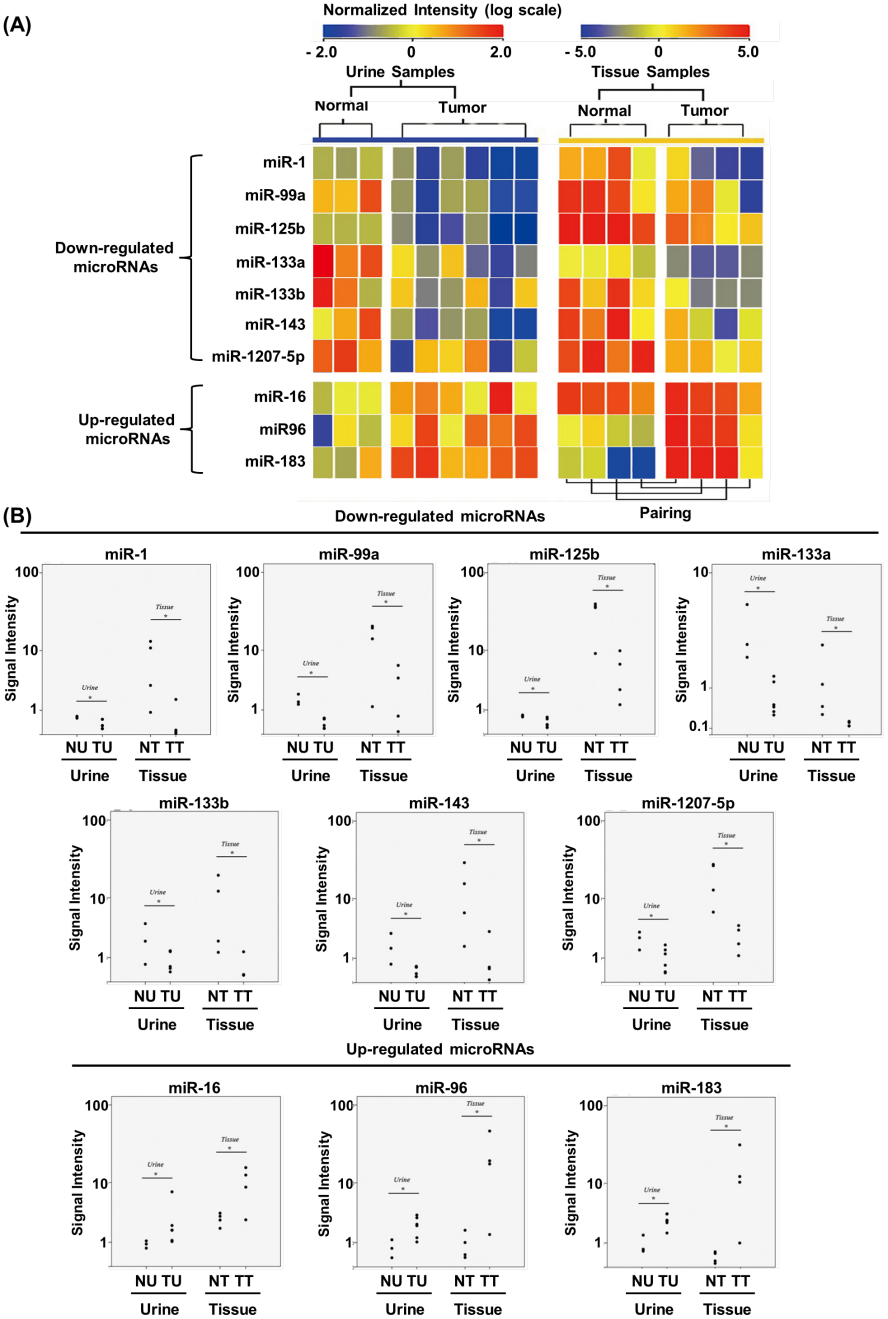
Results of microRNA microarray analysis. (A) Heat map of the levels of 10 selected microRNAs in nine urine supernatant samples and four pairs of tissue samples. The total RNA was extracted and subjected to microRNA microarray analysis using an Agilent Human microRNA Microarray Chip. The signal intensity of each spot was median-normalized and further normalized by linear regression. The normalized signal intensities for each microRNA in the urine supernatant and tissue samples of UCB patients and controls are presented. (B) Scatter plots of the normalized signal sensitivities of the 10 selected microRNAs. A Mann-Whitney U test was conducted to compare the levels of microRNAs in the urine supernatant of UCB patients (TU) (n = 6) and controls (NU) (n = 3), and a paired Mann-Whitney U test was conducted to compare the tumors (TT) (n = 4) and their corresponding normal mucosa tissues (NT) (n = 4) in the UCB patients. A significance level of 0.01 was used for all of the comparisons. Asterisks (*) indicate a statistically significant difference (p<0.01).

Urine supernatants from six UCB patients and three normal controls together with four pairs of UCB tissues and their corresponding normal bladder mucosal tissues were subjected to microRNA microarray analysis. The patient characteristics are shown in Table 1. To determine the presence and absence of microRNAs in the samples, a threshold signal was defined as the average background plus three standard deviations. Signals above this threshold were considered a “presence” and signals below the threshold were considered an “absence.” After global normalization, 117±63.8 out of 866 human microRNAs were detected in the urine supernatants and 314±83.4 microRNAs were detected in the bladder tissues. There was no significant difference in the total number of microRNAs detected between the urine supernatants from cancer patients and controls and between the cancer and control tissues. Comparing the profiles in the urine supernatants of the UCB patients with those of the normal controls, 39 microRNAs showed differential expression (Mann-Whitney test, p<0.05; fold differences >1.60). There were 78 differentially expressed microRNAs between the cancer and normal tissues (paired Mann-Whitney test, p<0.05; fold differences >2.0). Ten microRNAs were commonly dysregulated in both the urine supernatants and tissue samples (Figure 1 and Figure S1). Of these, the levels of microRNA-1, microRNA-99a, microRNA-125b, microRNA-133a, microRNA-133b, microRNA-143 and microRNA-1207-5p significantly decreased and those of microRNA-16, microRNA-96 and microRNA-183 increased in the UCB samples (Figure 1).

**Table 1:** Patient characteristics.

Characteristics	microRNA microarray			qRT-PCR			qRT-PCR
	Tissues (TT and NT)	Urine supernatant		Tissues (TT and NT)	Urine supernatant		Urine supernatant - pre- and post-operative
		TU	CU		TU	CU	
**Total number**	4 pairs	6	3	18 Pairs	50	21	20 pairs
**Median age**	74	71	62	71	75	65	75
**(years old)**	(58-81)	(58–81)	(61–70)	(45–88)	(43–85)	(32–87)	(43–85)
**Gender**							
Male	3	5	2	17	10	13	14
Female	1	1	1	1	40	8	6
**Stage**							
Superficial	2	2	-	8	41	-	17
Invasive	2	4	-	10	9	-	3
**Grade**						-	
Low grade (G1)	1	1	-	2	15	-	5
High grade (≥G2)	3	5	-	16	35	-	15
**Tumor status**						-	
New case	3	3	-	12	27	-	9
Recurrent case	1	5	-	6	23	-	11

TT: cancer tissue; NT: normal mucosa tissue; TU: urine supernatant from tumor patient; CU: urine supernatant from control.

### Validation of the 10 selected microRNAs in independent cohorts of tissue and urine supernatant samples

**Figure 2:**
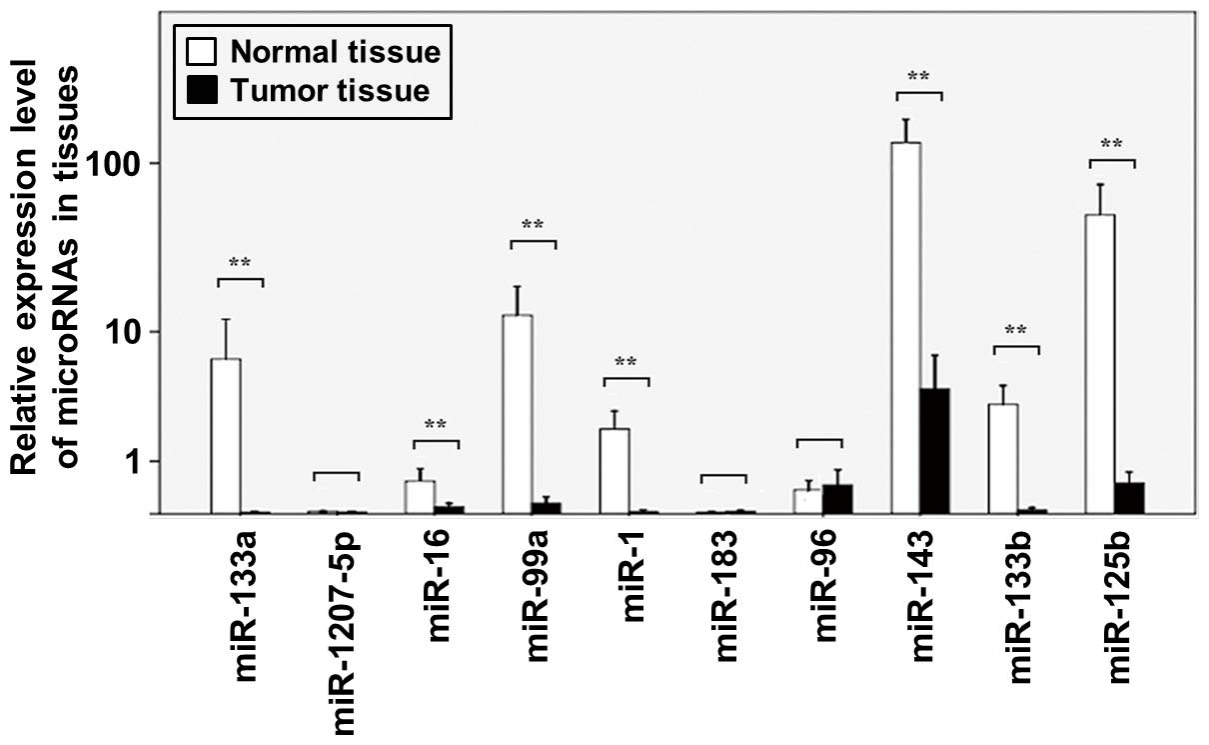
Validation study of the down-regulated expression of 10 selected microRNAs in bladder cancer tissues by RT-qPCR. The total RNA was extracted from 18 pairs of bladder cancer tissues and their corresponding normal adjacent mucosa tissues. The levels of these microRNAs were quantified by RT-qPCR in duplicate. The relative expression levels (2-ΔCq) are presented. A paired Mann-Whitney U test was conducted to compare the tumors and their corresponding normal mucosa tissues in UCB patients. Columns, Means; Bars, S.D.; n = 18. ** denotes p<0.001.

To validate the microarray results, the levels of the 10 selected microRNAs were quantified by RT-qPCR in another 18 pairs of bladder cancer and corresponding normal mucosa tissues (Table 1). Of these, the differential expression of six microRNAs between cancer and normal tissues was validated (Wilcoxon signed-rank two-related-samples test, p<0.05) (Figure 2). MicroRNA-1 was down-regulated in all 18 pairs of cancer tissues (p<0.01) and 17 out of 18 pairs showed down-regulation (p<0.01) in the remaining five microRNAs (microRNA-99a, microRNA-125b, microRNA-133a, microRNA-133b and microRNA-143). MicroRNA-1, microRNA-99a, microRNA-125b, microRNA-133a, microRNA-133b and microRNA-143 were down-regulated 266.87±2.06, 130.69±2.30, 49.52±3.39, 263.20±1.99, 261.38±2.11 and 67.18±1.83 times over in the cancer tissues, respectively.

**Figure 3:**
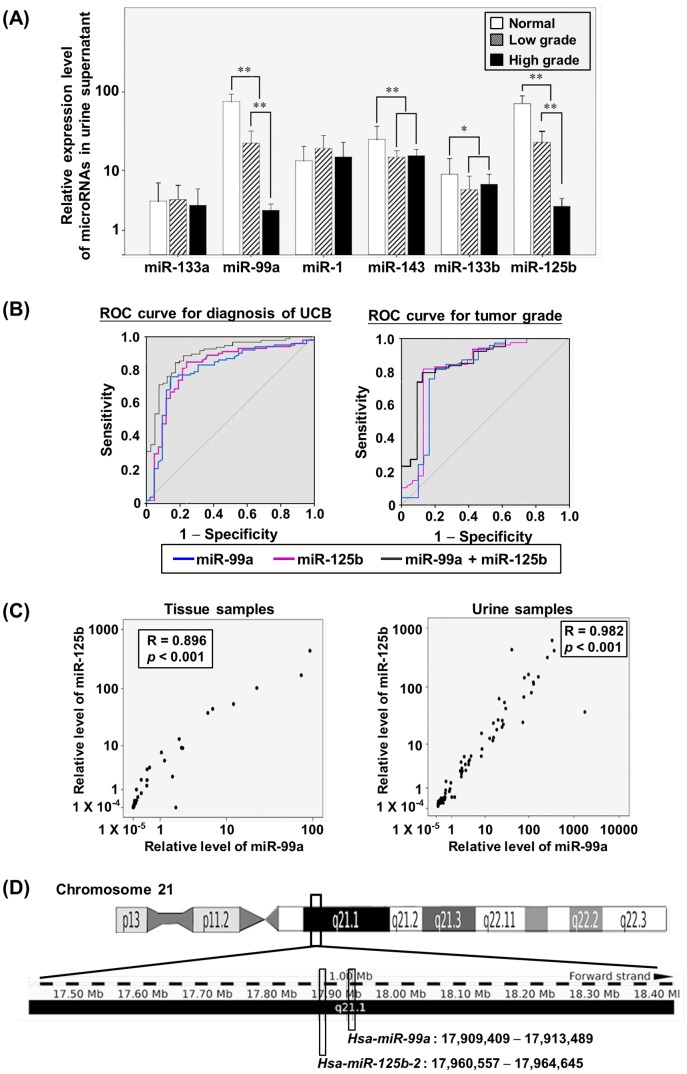
Expression of microRNAs in the urine supernatant of UCB patients and normal controls. The total RNA was extracted from the urine supernatants of UCB patients with low-grade (n = 15) (shaded bar) or high-grade cancers (n = 35) (black bar) and normal controls (n = 21) (white bar). The levels of six microRNAs in the samples were quantified by RT-qPCR in duplicate. (A) The relative levels (2-ΔCq) of microRNA-133a, microRNA99a, microRNA-1, microRNA-143, microRNA-133b and microRNA-125b in the urine supernatant samples are presented. A Mann-Whitney U test was conducted to compare the UCB patients and normal controls. Columns, Means; Bars, S.D.; ** denotes p<0.01; * denotes p<0.05. (B) ROC curves of microRNA-99a alone (blue line), microRNA-125b alone (red line) and microRNA-99a and microRNA-125b in combination (black line) for the diagnosis of UCB (left) and discrimination between low- and high-grade tumors (right). The cut-off points were set and the AUC values determined based on Youden's Index. (C) Results of a Pearson correlation analysis of the levels of microRNA-99a and microRNA-125b in the tissues and urine supernatant samples. Scatter plots of the relative levels of these two microRNAs in the samples are presented. The Pearson correlation coefficient R was calculated via a Pearson correlation analysis and the p-value was determined. (D) Chromosomal regions of the genes (*hsa-miR-99a* and *hsa-miR-125b-2*). Human Genome Reference Consortium GRCh37 Patch Release 12 assembly was used. *Hsa-miR-99a* is located at chromosome 21:17,909,409–17,913,489, and *hsa-miR-125b-2* is located at chromosome 21:17,960,557–17,964,645.

The discrimination power of the six validated microRNAs in the urine supernatants was then assessed using 71 urine supernatant samples, including 50 samples from bladder cancer patients (15 cases of low-grade cancer and 35 cases of high-grade cancer) and 21 samples from controls (Table 1). In this expanded set of samples, the urine supernatants of the UCB patients possessed lower levels of microRNA-99a (p<0.01), microRNA-125b (p<0.01), microRNA-133b (p<0.05) and microRNA-143 (p<0.01) than that of the controls (Figure 3A), but not of microRNA-133a and microRNA-1.

### Development of cell-free urinary microRNA models for the detection of UCB and the discrimination of tumor grades

The ROC curve was determined to evaluate the diagnostic strength of these microRNAs in detecting UCB. High area under the curve (AUC) values were found for microRNA-99a (0.800, ranging from 0.715 to 0.886) and microRNA-125b (0.813, ranging from 0.729 to 0.897) (Figure 3B), whereas microRNA-133b and microRNA-143 exhibited low diagnostic strength with AUC values around 0.6. To further determine the diagnostic significance of microRNA-99a and microRNA-125b, related cut-off points were determined from the ROC curves based on Youden's Index rule. The cut-off points for the normalized expression levels of microRNA-99a and microRNA-125b were 2^2.18^ and 2^4.1^, respectively. A patient with a urine supernatant whose microRNA level was lower than or equal to the cut-off point was considered a cancer case, and considered normal if the level was higher than the cut-off point. According to this system, the sensitivity, specificity, positive prediction value (PPV), negative prediction value (NPV), positive likelihood (LR+) and negative likelihood (LR-) for microRNA-99a were 78.0%, 85.7%, 92.7%, 61.0%, 5.46 and 0.26, respectively, and for microRNA-125b were 84.8%, 76.2%, 89.3%, 68.1%, 3.57 and 0.20, respectively (Table 2). To achieve better diagnostic performance, a combination of the expressions of microRNA-99a and microRNA-125b was subjected to a bivariate logistic regression. The CI performed better than the use of each microRNA alone. The AUC value increased to 0.876. The CI formula was CI = 1/{1+exp[−(2.332+0.425 xΔCq_microRNA-125b_+0.069 xΔCq_microRNA-99a_)]}. When the cut-off point was set at 0.6244 based on Youden's Index rule, the prediction system improved with an increased sensitivity (86.7%) and NPV (71.4%) and a decreased LR- (0.16), with comparable values for the specificity, PPV and LR+ (Table 2).

**Table 2:** Diagnostic strength of microRNA-99a alone, microRNA-125b alone and microRNA-99a and microRNA-125b in combination for the detection of bladder cancer and their significance in discriminating tumor grades.

	Diagnosis of UCB			Discrimination of tumor grades		
	microRNA-99a alone	microRNA-125b alone	microRNA-99a * and microRNA-125b	microRNA-99a alone	microRNA-125b alone	microRNA-99a# and microRNA-125b
**AUC**	0.800 (0.715∼0.886)	0.813 (0.729∼0.897)	0.876 (0.809∼0.942)	0.791 (0.673∼0.908)	0.819 (0.712∼0.926)	0.831 (0.770∼0.951)
**Cut-off point**	REL: 2^2.18^	REL: 2^4.1^	CI: 0.6244	REL: 2^0.91^	REL: 2^1.71^	CI: 0.7398
**Sensitivity**	78.0%	84.8%	86.7%	74.2%	81.4%	79.4%
**Specificity**	85.7%	76.2%	81.1%	83.3%	87.0%	88.0%
**PPV**	92.7%	89.3%	91.8%	91.5%	93.4%	94.7%
**NPV**	61.0%	68.1%	71.4%	58.1%	67.0%	61.1%
**+LR**	5.46	3.57	4.58	4.46	6.11	6.61
**-LR**	0.26	0.20	0.16	0.31	0.21	0.23

*Formula of CI for bladder cancer diagnosis: CI = 1/{1+exp[−(2.332+0.425 xΔCt_microRNA−125b_+0.069 xΔCt_microRNA-99a_)]}.

# Formula of CI for tumor grading: CI = 1/ {1+exp [−(1.742 +0.0295 xΔCt_microRNA−125b_ +0.496 xΔCt_microRNA-99a_)]}.

The clinical relevance of the four microRNAs selected to discriminate low-grade from high-grade cancers was also assessed. The levels of urinary microRNA-99a and microRNA-125b were significant lower in patients with high-grade cancers (G2 and G3) than in those with low-grade cancers (G1) (Mann-Whitney U test, p<0.01). The levels of microRNA-133b and microRNA-143 showed no tumor grade discrimination value (Figure 3A). The AUC values differentiating between high- and low-grade cancers for microRNA-99a and microRNA-125b were 0.819 and 0.791, respectively (Figure 3B). The cut-off points of the normalized levels of microRNA-99a and microRNA-125b for tumor grading were 2^1.71^ and 2^0.91^, respectively. Based on these cut-off points, the system using the level of microRNA-99a exhibited a sensitivity of 74.2%, a specificity of 83.3%, a PPV of 91.5%, an NPV of 58.1%, an LR+ of 4.46 and an LR- of 0.31. In the case of microRNA-125b, these values were 81.4%, 87.0%, 93.4%, 67.0%, 6.11 and 0.21, respectively, for discriminating tumor grades (Table 2). When combining the levels of microRNA-99a and microRNA-125b in the system, the CI formula became CI = 1/{1+exp[-(1.742 +0.0295xΔCq_microRNA-125b_ +0.496xΔCq_microRNA-99a_)]}, with 0.7398 as the cut-off point. The sensitivity, specificity, PPV, NPV, LR+ and LR- were 79.4%, 88.0%, 94.7%, 61.1%, 6.61 and 0.23, respectively (Table 2). All of these values were either comparable with or lower than those for the systems using either microRNA-99a or microRNA-125b alone. Taken altogether, the system using microRNA-125b alone performed better at differentiating between high- and low-grade UCB.

### Correlation between expressions of microRNA-99a and microRNA-125b in urine supernatants and bladder cancer and control tissues

Via a Pearson correlation analysis, the microRNA-99a and microRNA-125b levels in both the tissues and urine supernatants of the patients and controls were highly correlated (R = 0.896, p<0.001 for the tissue samples; R = 0.982, p<0.001 for the urine supernatant samples) (Figure 3C), suggesting that their expression may have been co-regulated. In fact, these genes were clustered at the q21.1 region of chromosome 21 (Figure 3D).

### Restoration of microRNA-99a and microRNA-125b expressions in post-operative patients

**Figure 4:**
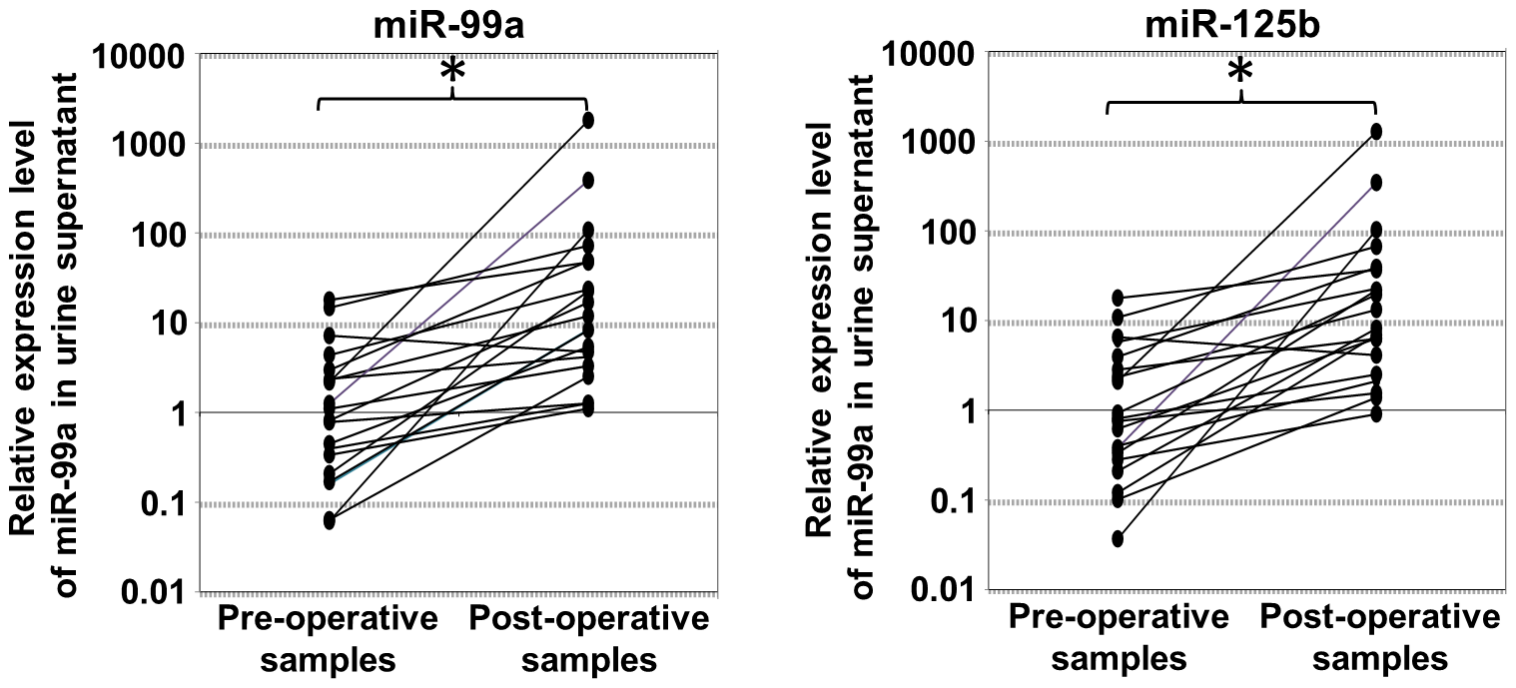
Restored expression of microRNA-99a and microRNA-125b in the urine supernatant of UCB patients after transurethial resection. Urine samples were collected from 20 UCB patients before (pre-operative) and after (post-operative) transurethial resection. The total RNA was extracted from the urine supernatants and subjected to RT-qPCR to quantify the levels of microRNA-99a (left) and microRNA-125b (right). A Wilcoxon signed-rank two-related-samples test was conducted to compare the levels of microRNA-99a and microRNA-125b between the pre- and post-operative samples. * denotes p<0.01.

To demonstrate the crucial link between bladder cancer status and decreased microRNA-99a and microRNA-125b levels in the urine supernatants, these two microRNAs were quantified in the urine supernatants of pre-operative and post-operative patients. Twenty patients were recruited (Table 1). Post-operative urine was collected 4 weeks after a transurethral resection. After the resection, the patients were able to restore the microRNA-99a and microRNA-125b levels in the urine supernatant (Wilcoxon signed-rank two-related-samples test, p<0.01) (Figure 4) to levels comparable with those of the normal control samples (Figure S2).

## Discussion

This study developed an effective cell-free urinary microRNA-based model for detecting UCB with good discriminating power (AUC  = 0.876) and high sensitivity (86.7%) and specificity (81.1%). The feasibility of using urinary microRNAs as bladder cancer diagnostic biomarkers was previously investigated. The ratio of microRNA-126 to microRNA-152 in urine was studied to detect UCB at a sensitivity of 82% and a specificity of 72%, with an AUC value of 0.768 [7]. By studying the expression levels of microRNA-96 and microRNA-183 in urine sediment, tests using these two microRNA biomarkers independently showed sensitivities of 71% and 74%, specificities of 89% and 77% and AUC values of 0.8331 and 0.817, respectively [18]. Using the CI of the microRNA-99a and microRNA-125b levels in urine supernatant here significantly improved the effectiveness of the diagnostic test for UCB detection. Moreover, the diagnostic performance was better than that of commercially available urine-based biomarkers (Table S1).

Unlike mRNA, which is vulnerable to environmental RNase, microRNAs are relatively stable in clinical samples. MicroRNAs remain intact even under unfavorable conditions such as high temperatures, extreme pH levels and 10 freeze-thaw cycles [6]. Moreover, they are readily detected in human plasma or serum by RT-qPCR, even at very low levels [7]–[8]. Tumor-associated changes to microRNA expression profiles in the circulation system have provided a new approach for cancer diagnosis and prognosis. In 2008, Lawrie et al. [19] reported that an elevated microRNA-21 serum level was associated with the relapse-free survival of patients with diffuse large B-cell lymphoma. Ng et al. [20] demonstrated the diagnostic significance of plasma microRNAs in colorectal cancer screening. These studies indicated the potential of circulating microRNAs in serum/plasma as biomarkers for cancer detection. In addition to serum/plasma, microRNAs are very stable in urine [17]–[18] and thus the detection of intact microRNAs in urine is promising. Wang et al. [21] identified cell-free urinary microRNA-146a and microRNA-155 as effective diagnostic biomarkers for systemic lupus erythematosus, implicating the clinical significance of cell-free urinary microRNAs in disease diagnosis and possibly prognosis. Although the yield of microRNAs from urine supernatant is relatively low compared with those from whole urine or urine sediment, urine supernatant is considered the best choice as a biomarker for disease diagnosis and prognosis [21]. Comparing the usefulness of urinary DNA in supernatant and sediment for UCB detection, Szarvas et al. [11] simultaneously analyzed urinary DNA in supernatants and sediment in patients and compared them to controls. Their results indicated that the urine supernatants yielded higher sensitivity than the sediment [11]. This was probably related to the contamination of blood cells in the urine samples of haematuria patients. The contaminated blood cells may have altered the urinary microRNA/DNA profiles in the samples and thus interfered with the cancer detection classification. In contrast, even the urine supernatants from patients with gross haematuria were relatively more homogeneous without interference due to the contaminated blood cells in the microRNA profiles. Therefore, urine supernatants may provide more convenient, reliable specimens for cancer diagnosis and disease monitoring.

Urinary microRNA profiles are subjected to various modifications such as the direct release of microRNAs from cancer tissues, immune responses to cancer and blood cells from haematuria patients. A feasibility study involving a candidate microRNA approach by qPCR was conducted to examine a small panel of microRNAs in the urine supernatants of bladder cancer patients [22]. Here, comprehensive microRNA profiles were obtained with microRNA microarrays of cell-free urine supernatants from UCB patients and controls who were confirmed to have no cystological abnormalities or other concurrent malignancies, and were compared to identify differentially expressed cell-free urinary microRNAs for UCB. The microRNAs were made a focus due to their direct release from cancer tissue and the levels of these candidates in cancer tissues and their normal counterparts were compared to validate the tissues. The microRNAs identified are probably functionally involved in UCB development and/or disease progression. They can be used as biomarkers for detection and disease monitoring. The microRNA microarray analysis results indicated the presence of more than 100 microRNAs in the urine supernatants. Among these, there were more down-regulated microRNAs than up-regulated microRNAs in the cancer patients' urine supernatants. This finding was similar to a previous finding that the majority of differentially expressed microRNAs in bladder cancer tissues were down-regulated [23]. These down-regulated microRNAs may play tumor suppressive roles in carcinogenesis [24]. The altered expression of six microRNA candidates identified by comparisons of the urinary cell-free microRNA profiles of UCB patients and controls were also observed in bladder tumor tissue in. Using an independent set of urine samples, the down-regulated expressions of four microRNAs were confirmed. As tumor tissue can directly secrete microRNAs into a patient's plasma [25], it is likely that urinary microRNAs are originally secreted by bladder cancer tissues and/or released from the necrotic/apoptotic malignant and even non-malignant epithelial cells, molding the microRNA profiles of urine supernatants. Thus, the expression patterns of various microRNAs in bladder cancer cells could be reflected by their urine supernatant profiles. However, not all of the differentially expressed microRNAs in cancer tissues can be used as urinary diagnostic biomarkers for UCB. The expressions of microRNA-1 and microRNA-133a were previously reported to be down-regulated in bladder cancer tissues and showed functional significance in carcinogenesis via targeting TAGLN2 in the cancer cells [26]. In the present study, their levels in the urine supernatant were examined, but no statistically significant difference was shown between the cancer patients and controls. This may imply other confounding factors such as the release of microRNAs from normal urinary epithelial cells and immune cells molding the microRNA profile in the urine supernatants to mask the differential expression of some cancer-related microRNAs.

Of the four microRNAs, microRNA-99a and microRNA-125b had the largest AUC values in the ROC curves for detection and could function as diagnostic biomarkers for bladder cancer. They were also found to be more significantly down-regulated in high-grade UCB than in low-grade UCB. After a complete transurethral resection of the tumors, the levels of previously down-regulated microRNA-99a and microRNA-125b in the urine supernatants increased, implicating a strong association between their levels and the patients' tumor statuses. This further supports their potential roles as diagnostic biomarkers for UCB and surveillance biomarkers for monitoring tumor recurrence. Based on these models, when a patient has a positive urine supernatant test result with a CI larger than the cut-off (0.6244), he or she probably has bladder cancer, with an accuracy rate of 91.8%. If the normalized expression level of microRNA-125b is lower than 2^1.71^, there is a 93.4% chance that the patient has high-grade cancer. However, because the negative predicting value was 71.4% in this study, negative results cannot be used to rule out the possibility of bladder cancer in patients.

The present study showed that the levels of microRNA-99a and microRNA-125b in the urine supernatant of UCB patients and controls were highly correlated with those in the tissues. In fact, the two genes (*hsa-miR-99a* and *hsa-miR-125b-2*) are located in intron 6 of the long intergenic non-protein coding RNA 478 (*LINC00478*) in chromosome 21. Their expressions are probably co-regulated by the promoter that controls the transcription of the *LINC00478* gene. The underlying mechanism for the down-regulation of these two microRNAs in UCB may be related to the frequently observed loss of genomic 21q in UCB. Tzai et al. [27] reported a frequent allelic loss of 36.6% in the long arm of chromosome 21, where the two genes are located, in UCB. In addition, patients with Down syndrome exhibited a decreased risk of dying from urological neoplasms, suggesting the presence of genes in chromosome 21 suppressing urological cancer tumors [28].

MicroRNA-99a and microRNA-125b have been demonstrated to have tumor-suppressive roles in various human cancers [29]-[34]. In prostate cancer, microRNA-99a suppresses the expression of prostate-specific antigens and prostate cancer cell proliferation by interfering with the expression of two chromatin remodeling factors, SMARCA5 (SWI/SNF-related matrix-associated actin-dependent regulator of chromatin subfamily A member 5) and SMARCD1 (SWI/SNF-related matrix-associated actin-dependent regulator of chromatin subfamily D member 1), and the growth regulatory kinase mTOR (mammalian target of rapamycin) [29]. In fact, a high expression of phosphorylated mTOR was found in 74% of muscle-invasive urothelical carcinomas, in association with increased pathological stages and decreased disease-related survival [30]. The inhibition of mTOR was found to decrease *in vitro* and *in vivo* bladder cancer cell growth [30]. As microRNA-99a targets mTOR in prostate cancer cells [29], the down-regulation of this microRNA in UCB demonstrated in the present study may abolish the tumor-promoting effects of mTOR in the cells, resulting in cancer development. However, further demonstration of the regulation of mTOR by microRNA-99a in bladder cancer cells is required. Although the data of the present study indicated a down-regulation of microRNA-99a expression in both the urine supernatants and tumor tissues of UCB patients, one report showed elevated microRNA-99a in noninvasive but not invasive lesions [31]. A down-regulation of microRNA-125b expression has been frequently observed in various tumors such as hepatocellular carcinoma HCC [32], osteosarcoma [33] and bladder cancer [34]. The ectopic expression of microRNA-125b in low-expressing cancer cells has been found to significantly decrease proliferation and promote apoptosis by targeting Bcl-2 in HCC cells [35], and to affect the proliferation and migration in osteosarcoma cell lines via the suppression of STAT3 expression in the cells [33]. In bladder cancer, microRNA-125b inhibited colony formation in an *in vitro* cell model and *in vivo* tumor development in nude mice by targeting E2F3, which frequently showed overexpression in bladder cancer and had an expression inversely correlated with that of microRNA-125b [34]. Taken together, aberrant expressions of microRNA-99a and microRNA-125b may be involved in bladder carcinogenesis and even disease progression. Further investigations that elucidate the mechanism underlying how these two microRNAs participate in bladder cancer development and progression are required. The present study found that microRNA-99a and microRNA-125b can be used as effective biomarkers for UCB detection and disease monitoring. They can also function as therapeutic agents for UCB treatment in patients.

## Conclusions

The results of this study revealed the unique microRNA expression signature in urine supernatants and tumor tissues of bladder cancer patients for the development of molecular diagnostic tests by microRNA microarray and RT-qPCR. The differential expression of microRNA-99a and microRNA-125b in an expanded patient cohort was verified. An effective cell-free urinary model using a combined index of the microRNA-99a and microRNA-125b levels for bladder cancer detection with good discriminating power, high sensitivity and high specificity was developed. The diagnostic significance of these two microRNAs was further confirmed by the reversals of the altered expression in patients after tumor resection. It was found that microRNA-99a and microRNA-125b can be used as effective biomarkers for UCB detection and disease monitoring. They can also function as therapeutic agents for UCB treatment in patients.

## Supporting Information

Figure S1 Workflow of the study of microRNA profiles in urine supernatant and cancer and normal tissues of bladder cancer patients and controls. microRNA profiles were determined by microRNA microarray for identification of differentially expressed microRNAs. By RT-qPCR, the differential expression of the selected microRNAs was validated in the tissue samples of the patients and also in the urine supernatant samples of the expanded patient cohort for development of models for detection of bladder cancer. To determine the link between tumor status and the differential expression, the relative levels of the selected microRNAs in urine supernatant of pre-operative and post-operative patients was determined by RT-qPCR and compared.
(TIF)

Figure S2 Expression of microRNA-99a and microRNA-125b in urine supernatant of UCB patients and normal controls. Urine samples were collected from 20 UCB patients before (pre-operative) and after (post-operative) transurethial resection. Total RNA was extracted from supernatant of pre- and post-operative urine samples of UCB patients (n = 20) and also that of normal controls (n = 21). The levels of these two microRNAs in the samples were quantified by RT-qPCR in duplicate. Relative levels (2-ΔCq) of microRNA-99a and microRNA-125b in the urine supernatant samples were presented. Wilcoxon signed-rank 2 related samples test was used for the comparisons of the levels of microRNA-99a and microRNA-125b between pre-operative and post-operative samples. For the comparisons between the post-operative urine samples of UCB patients and normal controls, Mann-Whitney U test was used. Columns, Means; Bars, S.D.; * denotes *p*<0.01 for Wilcoxon signed-rank 2 related samples test; # denotes *p*>0.05 for Mann-Whitney U test.
(TIF)

Table S1 Median sensitivity and specificity of cytology and other urine-based markers for the detection of bladder cancer.
(DOCX)
