# Supplementary figures and images for: Cell-Free Urinary MicroRNA-99a and MicroRNA-125b Are Diagnostic Markers for the Non-Invasive Screening of Bladder Cancer
Source: PLoS One. 2014 Jul 11;9(7):e100793. doi: 10.1371/journal.pone.0100793 (PMC4094487; doi:10.1371/journal.pone.0100793)

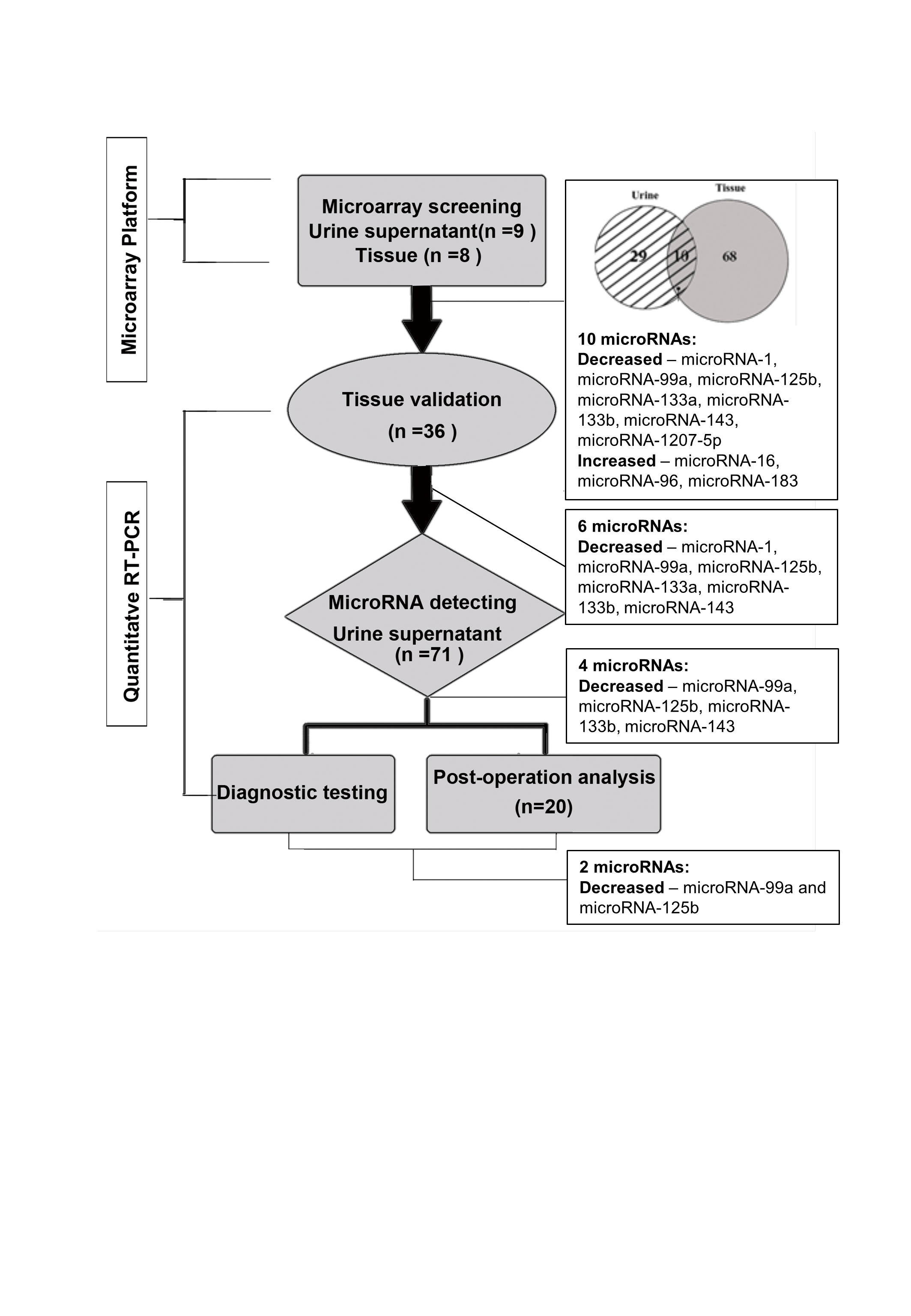

Supplement: Figure S1 — Workflow of the study of microRNA profiles in urine supernatant and cancer and normal tissues of bladder cancer patients and controls. microRNA profiles were determined by microRNA microarray for identification of differentially expressed microRNAs. By RT-qPCR, the differential expression of the selected microRNAs was validated in the tissue samples of the patients and also in the urine supernatant samples of the expanded patient cohort for development of models for detection of bladder cancer. To determine the link between tumor status and the differential expression, the relative levels of the selected microRNAs in urine supernatant of pre-operative and post-operative patients was determined by RT-qPCR and compared. (TIF) [file pone.0100793.s001.tif]
