# Supplementary material for: Cell-Free Urinary MicroRNA-99a and MicroRNA-125b Are Diagnostic Markers for the Non-Invasive Screening of Bladder Cancer
Source: PLoS One. 2014 Jul 11;9(7):e100793. doi: 10.1371/journal.pone.0100793 (PMC4094487; doi:10.1371/journal.pone.0100793)
Supplement: Table S1 — Median sensitivity and specificity of cytology and other urine-based markers for the detection of bladder cancer. (DOCX) [file pone.0100793.s003.docx]

| **Supplementary Table S1: Median sensitivity and specificity of cytology and other urine-based markers for the detection of bladder cancer** | | | | | | | |
| --- | --- | --- | --- | --- | --- | --- | --- |
| Diagnostic tests | No. of Studies | | Sensitivity | | Specificity | |  |
| Cytology | 24 | | 41.65 | | 94.95 | |  |
| Hematuria Dipstick | 7 | | 50.4 | | 66.1 | |  |
| Bladder Tumor Antigen test (BTA) Stat | 8 | | 75.2 | | 77.45 | |  |
| Bladder Tumor Antigen test (BTA) Trak | 3 | | 75.6 | | 73 | |  |
| ImmunoCyt | 6 | | 79.65 | | 74.4 | |  |
| uCyt+ | 6 | | 85.05 | | 72.75 | |  |
| NMP22 Bladder Cancer Test | 11 | | 73.5 | | 77.25 | |  |
| NMP22 BladderChek | 5 | | 57.1 | | 73 | |  |
| UroVysion/FISH | 7 | | 81 | | 77.7 | |  |
| **Reference**: Budman LI, Kassouf W, Steinberg JR (2008) Biomarkers for detection and surveillance of bladder cancer. Can Urol Assoc J 2(3):212-221. | | | | | | | |
|  | |  | |  | |  | |
